# Genital self-mutilation in a schizophrenic patient: A case report

**DOI:** 10.1016/j.ijscr.2025.111587

**Published:** 2025-06-30

**Authors:** Salim Ouskri, Adam El Aboudi, Idriss Ziani, Imad Boualaoui, Hachem El Sayegh, Yassine Nouini

**Affiliations:** Ibn Sina Hospital, Morocco

**Keywords:** Genital self-mutilation, Penile amputation, Microsurgical reimplantation, Ischemic necrosis

## Abstract

**Introduction:**

Genital self-mutilation is a rare but severe form of self-inflicted injury, most commonly associated with psychiatric disorders, particularly schizophrenia. It poses significant challenges due to the need for urgent urological intervention and psychiatric stabilization. While self-harming behaviors are frequent in psychiatric populations, complete genital mutilation remains an exceptionally rare event [1, 2].

**Case presentation:**

A 38-year-old schizophrenic male, previously well-managed under antipsychotic treatment, presented to the emergency department 1 h after a self-inflicted complete amputation of the penile glans during an acute psychotic episode. On examination, he was hemodynamically stable, with a clean transection, minor hesitation lacerations, and a cold but non-necrotic amputated glans. After emergency psychiatric evaluation and stabilization, he underwent microsurgical reimplantation involving arterial and venous anastomoses (dorsal penile artery and deep dorsal vein), urethral reconstruction, and soft tissue repair.

**Discussion:**

Despite initial postoperative improvement, progressive ischemia of the glans developed by day 4, leading to necrosis, necessitating revision surgery with debridement. Genital self-mutilation is significantly rarer than outward-directed aggression in schizophrenia. While microsurgical replantation can offer functional recovery, vascular complications remain common. Psychiatric stabilization is critical in preventing recurrence.

**Conclusion:**

Genital self-mutilation in schizophrenic patients represents a complex intersection of psychiatric crisis and urological emergency. Successful management requires a multidisciplinary approach, combining emergency surgery with psychiatric intervention to optimize outcomes and prevent future self-harm. Further research is needed to refine treatment protocols and long-term psychiatric follow-up strategies.

## Highlights

• Severe genital self-mutilation is rare and typically associated with schizophrenia.
• A schizophrenic patient amputated his glans; reimplantation was attempted in emergency.
• Postoperative glans necrosis occurred and required secondary surgical management.
• Urgent multidisciplinary care is vital; psychiatric follow-up is key to prevent recurrence.

## 1 Introduction

Genital self-mutilation is a rare but serious self-inflicted injury, most often associated with severe psychiatric disorders, particularly schizophrenia. These cases present complex challenges, requiring both urgent urological intervention and comprehensive psychiatric management. The act is typically driven by acute psychotic episodes, often involving delusions or hallucinations. While self-harming behaviors are not uncommon in psychiatric populations, complete genital mutilation remains an exceptionally rare occurrence. Large trauma series have reported only a handful of cases among thousands of admissions, highlighting its infrequency [1,2]. This article presents a case of genital self-mutilation in a schizophrenic patient, discussing its prevalence, underlying psychiatric and medical factors, diagnostic considerations, and the importance of a multidisciplinary treatment approach, including potential surgical reimplantation strategies.

This work has been reported in line with the SCARE criteria.

Kerwan A, Al-Jabir A, Mathew G, Sohrabi C, Rashid R, Franchi T, Nicola M, Agha M, Agha RA. Revised Surgical CAse REport (SCARE) guideline: An update for the age of Artificial Intelligence. Premier Journal of Science 2025:10;100079.

## 2 Case presentation

A 38-year-old male patient, diagnosed with schizophrenia four years prior, presented to the emergency department following a self-inflicted complete amputation of the penile glans. He had been managed effectively with a standard antipsychotic regimen, including risperidone 4 mg daily and aripiprazole 10 mg daily under regular psychiatric follow-up. The incident occurred during an acute manic-depressive episode (BDA), during which the patient used a sharp knife to sever the glans penis completely. He was brought to the hospital within 1 h of the injury (H1).

**Fig. 1:**
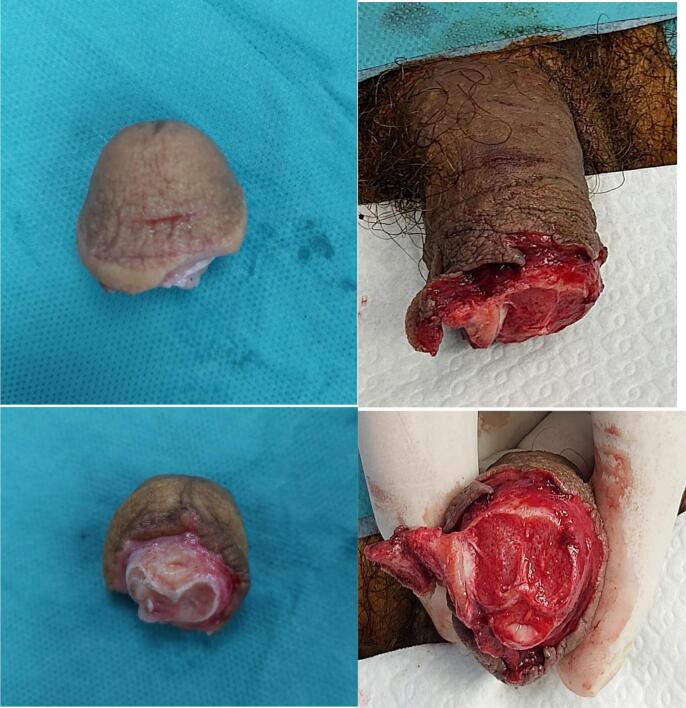
Sequence of images showing a case of self-inflicted penile amputation with Detached glans and clean transection.

Upon arrival, the patient was hemodynamically stable, with mild sheet-like bleeding from the erectile bodies noted at the injury site. Physical examination revealed a clean transection of the glans with minor signs of hesitation, evidenced by irregular, clumsy lacerations adjacent to the primary cut. The amputated glans was pale and cold but showed no signs of necrosis, consistent with the short ischemic time (Fig. 1). The severed segment was preserved in saline-soaked gauze and placed in an ice slush bath by emergency personnel.

**Fig. 2:**
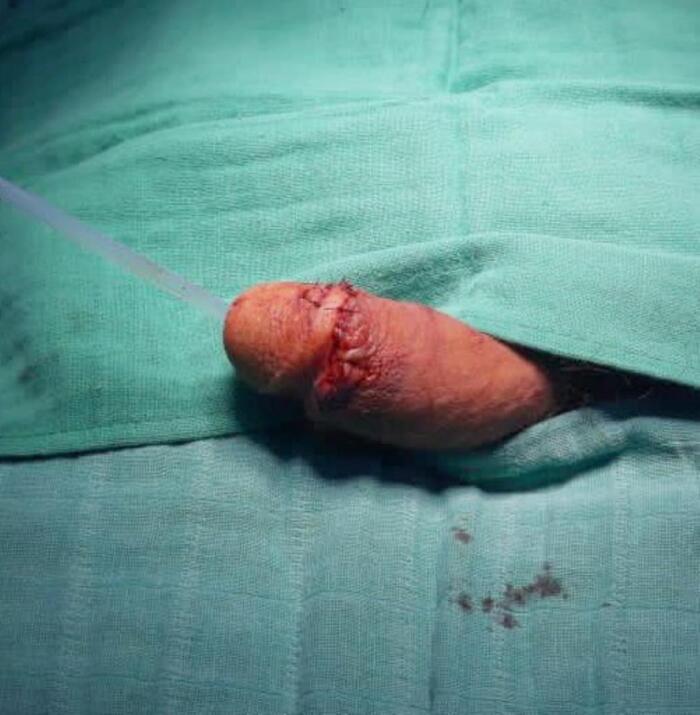
Postoperative view showing successful microsurgical replantation of the penile glans.

Following an urgent psychiatric evaluation confirming acute psychosis, the patient was sedated with intramuscular haloperidol 5 mg and lorazepam 2 mg to stabilize his mental state. Consent for surgical intervention was obtained from the family due to the patient's impaired capacity. He was immediately transferred to the operating theater for replantation. The procedure involved microsurgical anastomosis of the dorsal penile artery and deep dorsal vein to restore vascular supply. A termino-terminal anastomosis of the penile urethra was performed over an 18 Fr urinary catheter, followed by reapproximation of the tunica albuginea of the corpora cavernosa using interrupted sutures. Subcutaneous tissues and skin were closed with simple sutures (Fig. 2).

**Fig. 3:**
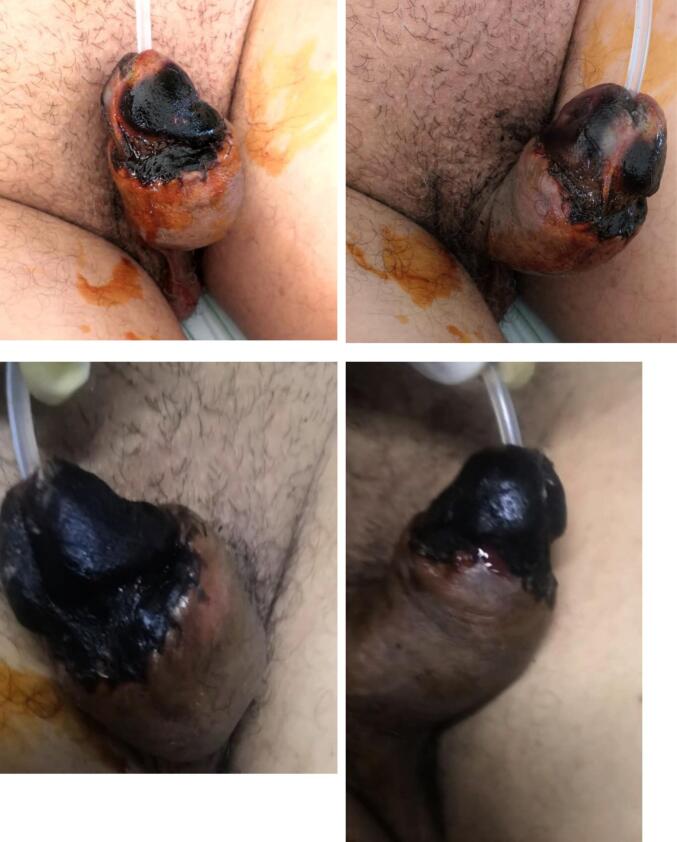
Sequential images showing the progression of ischemic necrosis after penile glans reimplantation.

The initial postoperative course showed promising recovery of the glans within the first 24 h. However, by postoperative day 4, progressive ischemic changes emerged, marked by darkening of the glans tissue, indicative of necrosis. The patient subsequently underwent a revision surgery for debridement of the necrotic tissue, regularizing the penile stump. Further management included psychiatric stabilization and wound care (Fig. 3).

## 3 Discussion

Genital self-mutilation is significantly less common than other forms of aggression or violence among psychiatric patients. While outward aggression affects up to 20–30 % of patients with schizophrenia during acute episodes, extreme self-inflicted injuries like penile amputation remain exceptionally rare. Morrison et al. reported that among 106 penile amputation cases, 55.8 % were self-inflicted, predominantly in psychotic contexts, yet still represented a small subset of self-harm behaviors [3]. Similarly, in the penile garroting series by Koushik et al., only one of three cases had a psychiatric background (depression), and none involved schizophrenia or complete amputation [4]. These findings reinforce the idea that genital self-mutilation is a unique and extreme manifestation of psychopathology, diverging from more common self-harming patterns.

Schizophrenia remains the principal underlying condition associated with genital self-mutilation. Greilsheimer and Groves, cited by Jezior et al., identified schizophrenia in 51 % of such cases, often linked to delusional ideation or command hallucinations [1]. Koushik et al. also cited cases from Jandou et al. implicating schizophrenia in severe genital trauma [4]. Other psychiatric comorbidities, including major depression (19 %) and personality disorders, play a secondary role [1]. These acts often occur in response to internally driven distortions, such as beliefs in bodily purification or response to divine command, as illustrated in the case of Raheem et al., where methamphetamine-induced psychosis triggered penile amputation [2].

The diagnostic process requires simultaneous somatic and psychiatric assessment. Urologically, the extent of vascular and urethral injury must be evaluated urgently to determine the feasibility of reimplantation. Grading systems such as that of Bhat et al. (cited in Koushik et al.) may guide initial classification [4]. Psychiatrically, structured clinical evaluations, including MMSE and full psychiatric assessment, are necessary to identify psychosis, delirium, or substance-induced episodes. Differentiation from traumatic or criminal injury remains critical, as the implications for management and long-term surveillance differ significantly.

Therapeutic management is based on an urgent multidisciplinary approach. When technically feasible, microsurgical reimplantation is the preferred option. In the series by Morrison et al., microsurgical techniques were used in 67 % of cases, with favorable urinary (97.4 %) and erectile (77.5 %) functional outcomes [3]. Raheem et al. describe similar reconstructive steps, including anastomosis of dorsal vessels and nerves, with acceptable sensory and erectile recovery [2]. Our approach followed these principles, with revascularization, urethral repair, and anastomosis of dorsal penile structures. Early evolution was initially favorable, yet the patient developed progressive ischemia and total necrosis of the glans by the fourth postoperative day, requiring revision surgery with debridement.

This unfavorable outcome highlights the persisting risk of early vascular compromise despite optimal ischemic conditions and successful anastomosis. Known complications of penile reimplantation include skin necrosis (54.8 %), venous congestion (20.2 %), urethral stricture (11 %), and urethrocutaneous fistula (6.6 %) [3]. Several hypotheses may explain such ischemic evolution, including unrecognized microthrombosis, vasospasm, or localized hypercoagulable states associated with acute psychosis. Current techniques do not always allow intraoperative prediction of these events. Adjunct tools such as indocyanine green angiography or intraoperative Doppler assessment may help evaluate perfusion more accurately, but their routine use in emergency penile replantation remains to be clarified.

Psychiatric stabilization is essential both for acute care and long-term prevention. In this case, initial sedation with haloperidol and lorazepam was followed by reintroduction of antipsychotic therapy under supervised psychiatric care. Previous studies have emphasized the importance of early and ongoing psychiatric support to limit the risk of recurrence [1,2,4]. Structured psychiatric follow-up, patient and family education, and therapeutic alliance are critical components in managing patients with high-risk psychotic disorders. In addition to pharmacological management, integration into psychosocial rehabilitation programs and systematic monitoring may reduce the likelihood of future self-injurious behavior.

Microsurgical replantation remains technically feasible up to 15–16 hour post-injury, particularly with optimal hypothermic preservation [1,3]. Favorable outcomes are associated with clean-cut injuries, early intervention, and successful vascular anastomosis. Morrison et al. report 68.4 % of patients achieving full sensation, 77.5 % erectile function, and 91.6 % satisfaction [3]. However, the variability of outcomes and the incidence of complications emphasize the need for careful intraoperative and postoperative surveillance. Jezior et al. noted improved long-term function with microsurgical techniques compared to non-microsurgical approaches, with better rates of normal erections (79 %) and preserved sensation (82 %) [1]. Despite these advancements, complications such as early ischemia remain a significant challenge, particularly in complex cases with psychiatric comorbidity.

## 4 Conclusion

Genital self-mutilation in schizophrenic patients, though rare compared to aggression, represents a critical intersection of urological emergency and psychiatric crisis, predominantly driven by acute psychosis. Effective management hinges on rapid diagnosis and a coordinated approach involving microsurgical replantation—yielding high success rates in sensation, urinary, and erectile function when criteria like short ischemia time and vessel integrity are met—and aggressive psychiatric stabilization to address underlying schizophrenia and prevent recurrence. Complications such as skin necrosis and urethral strictures remain challenges, yet outcomes are generally positive with modern techniques. Further research is warranted to standardize surgical protocols and enhance long-term psychiatric interventions for these complex cases.

## Consent

Written informed consent was obtained from the patient for publication and any accompanying images. A copy of the written consent is available for review by the Editor-in-Chief of this journal on request.

## Ethical approval

Ethical approval for this study was provided by the Ethical Committee of **IBN SINA** University Hospitals, Rabat, Morocco on 10/02/2025. Number of the decision is not delivered yet.

## Guarantor

Salim Ouskri

## Funding

No source of funding.

## Methods

This work has been reported in line with the SCARE criteria.

## Author contribution

Salim Ouskri - Urology Resident, IBN SINA HOSPITAL (Corresponding author)

Email: salim.ouskri@gmail.com

ADAM EL ABOUDI- Urology Resident, IBN SINA HOSPITAL

Email: elaboudiadam2@gmail.com

IDRISS Ziani - Urologist, IBN SINA HOSPITAL

Email: Idrissziani20@gmail.com

Imad Boualaoui - Urology Assistant Professor, IBN SINA HOSPITAL

Email: imadboualaoui@gmail.com

Hachem El Sayegh - Urology Professor, IBN SINA HOSPITAL

Email: hachemsayegh@yahoo.fr

Yassine Nouini - Urology Professor, IBN SINA HOSPITAL

Email: ynouini@yahoo.fr

## Conflict of interest statement

I declare no conflict of interest.
